# Testing for sensory threshold in drinking water with added calcium: a first step towards developing a calcium fortified water

**DOI:** 10.12688/gatesopenres.13361.2

**Published:** 2022-01-10

**Authors:** Gabriela Cormick, Natalia Matamoros, Iris B. Romero, Surya M. Perez, Cintia White, Dana Z. Watson, José M. Belizán, Miriam Sosa, M. Fernanda Gugole Ottaviano, Eliana Elizagoyen, Lorena Garitta

**Affiliations:** 1 Department of Mother and Child Health Research, Institute for Clinical Effectiveness and Health Policy (IECS-CONICET), Ciudad de Buenos Aires, 1414, Argentina; 2 Centro de Investigaciones en Epidemiología y Salud Pública (CIESP-IECS), Consejo Nacional de Investigaciones Científicas y Técnicas (CONICET), Ciudad de Buenos Aires, 1414, Argentina; 3 Departamento de Ciencias de la Salud, Universidad Nacional de La Matanza (UNLaM), San Justo, 1754, Argentina; 4 Instituto de Desarrollo E Investigaciones Pediátricas "Prof. Dr. Fernando E. Viteri" Hospital de Niños "Sor María Ludovica de La Plata (IDIP), Ministerio de Salud/Comisión de Investigaciones Científicas de La Provincia de Buenos Aires, La Plata, 1900, Argentina; 5 Departamento de Evaluación Sensorial de Alimentos (DESA), Instituto Superior Experimental de Tecnología Alimentaria (ISETA), 9 de Julio, Buenos Aires, Argentina; 6 Consejo Nacional de Investigaciones Científicas y Técnicas (CONICET), Buenos Aires, Argentina; 7 Comisión de Investigaciones Científicas de la provincia de Buenos Aires (CIC), Buenos Aires, Argentina

**Keywords:** Drinking water, calcium salts, survival analysis, triangle test, calcium inadequacy

## Abstract

**Background: **Food fortification is an effective strategy that has been recommended for improving population calcium inadequate intakes. Increasing calcium concentration of water has been proposed as a possible strategy to improve calcium intake. The objective of this study was to determine the sensory threshold of different calcium salts added to drinking water using survival analysis.

**Methods**: We performed the triangle test methodology for samples of water with added calcium using three different calcium salts: calcium chloride, calcium gluconate and calcium lactate. For each salt, a panel of 54 consumers tested seven batches of three water samples. Data were adjusted for chance and sensory threshold was estimated using the survival methodology and a discrimination of 50%.

**Results**: The threshold value estimation for calcium gluconate was 587 ± 131 mg/L of water, corresponding to 25% discrimination, for calcium lactate was 676 ± 186 mg/L, corresponding to 50% discrimination, and for calcium chloride was 291 ± 73 mg/L, corresponding to 50% discrimination.

**Conclusions**: These results show that water with calcium added in different salts and up to a concentration of 500 mg of calcium/L of water is feasible. The calcium salt allowing the highest calcium concentration with the lowest perceived changes in taste was calcium gluconate. Future studies need to explore stability and acceptability over longer periods of time.

## Introduction

Calcium intake is well below recommendations in most low- and middle-income countries, and in many countries calcium availability from foods does not cover the needs of their populations ^1– 5^. Appropriate calcium intake has shown many health benefits besides the prevention of osteoporosis such as reduction of hypertensive disorders in pregnancy, lower blood pressure, lower cholesterol values, lower blood pressure in children whose mothers were supplemented with calcium during pregnancy and prevention of recurrence of colorectal adenomas ^6– 11^.

Food fortification is an effective strategy that has been successfully used to reduce micronutrient deficiencies ^12^. Increasing the calcium concentration of water is a possible strategy to improve calcium intake ^13^. Although there are natural mineral waters with high calcium contents on the market, calcium concentration in tap water and commercially bottled water seems to be low in most parts of the world ^14– 17^. There are many advantages to using water as a fortification vehicle as it is universally consumed, calcium in water has good bioavailability, similar to that of milk, and it is consumed throughout the day, which also improves absorption ^18, 19^. Simulations of the impact of water supplemented with 500 mg of calcium on the calcium intake of populations of different countries with low calcium intake have shown an increase in the percentage of people reaching adequate intakes without exceeding the risk for excess, measured by the recommended upper limit for calcium ^13^.

Designing a strategy to increase the calcium concentration of drinking water first requires an exploration of the physicochemical changes and organoleptic properties of water with added calcium ^20, 21^. A first step is to define the type of salt and concentration at which the organoleptic characteristics are acceptable for consumers. Thresholds are useful measures for determining an individual’s or group’s average sensitivity to a tastant or odorant chemical. Sensory thresholds are often collected through ascending forced-choice methods, like three-alternative forced choice (3-AFC) ^22^ or triangle test ^23^. However, in methods of ascending concentrations a person may guess the correct answer by chance or might detect it at low concentrations, but fail after several steps due to fatigue or adaptation. The use of statistical survival analysis considers the group’s results of threshold data collected through the forced-choice method reducing the probability of chance in methods with consecutive correct answers ^24^.

The objective of this study was to determine the sensory detection threshold of different calcium salts added to drinking water using survival analysis.

## Methods

### Ethics statement

This study was approved by the ethical committee of the Posadas Hospital (ref: 318 EUPeSe/19). Participants in this study received oral and written explanations of the protocol and signed an informed consent form for participation and use of data.

### Selection of salts and concentrations

For this study, we selected three calcium salts suitable for human consumption, commonly used by the food industry and with a reported solubility allowing solutions of at least 500 mg of calcium per liter at room temperature (20°C). The solubility of these three salts theoretically allows solutions to be obtained that widely exceed this value. Calcium chloride dihydrate has solubility in water of 740 g/L at 20°C (up to 200,000 mg Ca/L), calcium gluconate monohydrate has a solubility in water of 32,7 g/L at 20°C (up to 2900 mg Ca/L), and calcium lactate pentahydrate has a solubility in water of 58 g/L at 20°C (7500 mg Ca/L) ^25– 27^. Calcium chloride dihydrate was purchased from Sigma-Aldrich Corporation, and meets analytical specifications of The European Pharmacopoeia (Ph. Eur.), Pharmaceutical Reference Standards (USP), FCC, E509, whereas calcium lactate pentahydrate and calcium gluconate Monohydrate were purchased from Surfactan, and both meet analytical specifications of USP and European Pharmacopoeia (EP).

The concentrations used to determine the sensory detection threshold of each calcium salt are shown in Table 1. They were defined taking into account the solubility of different calcium salts, previous studies performed in different countries showing that 500 mg of Ca/L increases the percentage of people reaching adequate intake without exceeding the recommended upper limit for calcium intake and a triangle test performed by an expert panel ^12^. This expert panel of six assessors were selected and trained following the guidelines of ISO 8586-1 ^28^, and had a minimum of 100 hours experience in discrimination and descriptive tests.

Using the triangle test, the expert panel compared concentration where no sensory differences were detected and concentrations where marked differences were detected (77 and 800 mg of Ca / L for calcium chloride, 107 and 820 mg of Ca / L for calcium lactate, and 107 and 863 mg of Ca / L for calcium gluconate) against water without added calcium to define the seven samples used in the sensory threshold test. Table 1

### Baseline water

All the concentrations prepared from the different calcium salts were tested against a reference bottled table water that is commonly consumed in the country and complies with the standards of the water cooperative in Argentina (Instituto verificador de elaboración de soda en sifones, IVESS) ^29^. According to the information provided by the cooperative, this water is filtered, dechlorinated, ozone purified tap water. The baseline bottled water had a calcium concentration of 27 mg/L and water hardness of 104 mg/L (CaCO _3_). This water also contained 32 mg/L of sodium, 10mg/L of nitrates, 38 mg/L of chlorides, 80 mg/L of alkalinity (CaC0 _3_) and 270 mg/L total dissolved solids.

### Sample preparation

Samples were prepared using the previously described baseline bottled table water. Solutions were prepared registering the weight of the salt added. The calcium concentration was measured in a sample of the final solution by atomic absorption spectroscopy at 422.7 nm (Varian AA 240FS) in an acetylene-air flame, the technique used was based on the Standard methods for the examination of water and wastewater ^30^. Table 1 shows the calcium concentrations tested for each salt.

**Table 1.** Calcium concentrations for each salt tested in the triangle test.

Samples	mg of calcium/L of water		
	Calcium gluconate monohydrate	Calcium lactate pentahydrate	Calcium chloride dihydrate
1	116	146	104
2	151	195	130
3	231	233	177
4	361	378	260
5	394	474	372
6	692	702	518
7	796	820	755

### Consumer panel

The panel was selected from a consumer´s database hold by the Departamento de Evaluación Sensorial de Alimentos- Instituto Superior Experimental de Tecnología Alimentaria (DESA-ISETA) from the city of 9 de Julio (Buenos Aires, Argentina). Individuals registered in the database have previously participated in consumer panel tests and agreed to be contacted by the institute for similar tests. Those who were aged 18 or older and reported drinking water every day were invited to participate.

The number of consumers was based on the requirements of the ISO 4120:2004 ^23^, for similarity tests. The parameters considered for this work were: β:5%; α :10%; Pd:30%; leading to a total of 54 consumers. The tests were conducted in the facilities of DESA-ISETA.

### Sensory methodology

We perform a triangle test to detect the threshold taste for each salt following the ISO/FDIS- 4120:2004 (E) ^23^. Before the triangle test the consumer panel received a short training session on the triangle test methodology and the procedures required to taste water samples. As part of the training, participants were asked to detect the odd sample of three 10 ml samples, two containing water and one water supplemented with 10 grams of sugar per liter.

After the training, threshold tests were carried out. In this test, a batch of three samples were presented simultaneously to the panelists, two samples were from the same concentration, and one is from a different concentration. There were six possible serving orders (AAB, ABA, BAA, BBA, BAB, ABB) which were counterbalanced across all panelists. Consumers were asked to taste each sample in the row (left to right) and to select the odd sample. Between batches, participants were asked to neutralize taste with mineral water and white bread.

Each consumer received seven batches of three solutions of 30 ml each. The tests were performed on different days to avoid tiredness or flavour carryover. For each salt, consumers tested three batches one day and the remaining four batches on a second day.

Samples were presented at room temperature (18–23°C) in similar polystyrene 70 mL cups coded with three-digit random to allow blinding, following sensory analysis protocols, where only participants are blinded to the samples ^31^.

### Statistical analysis

The statistical model developed by Hough *et al.* (2013) was applied to the data obtained in the triangle test ^24^. A random variable C is the salt concentration at which an assessor correctly discriminates a sample. The discrimination function D(c) can be defined as the probability of an assessor discriminating a sample before concentration c, i.e., D(c) = P(C ≤ c). For example, the log-normal distribution is expressed by:


$$\%\;Discri\min ation\;=\;\text{Φ}\left(\frac{\log(concentration)-\text{μ}}{\text{σ}}\right)x\;100\;(1)$$

and the Weibull distribution is expressed by:


$$\%\;Discri\min ation\;=\;\exp\left(-\exp\left(\frac{\log(concentration)-\text{μ}}{\text{σ}}\right)\right)x\;100\;(2)$$

Where, in Equation (1), Φ (.) is the cumulative normal distribution function and µ and σ are the model’s parameters.

In Equation (2), exp [ -exp, is the distribution function of the smallest extreme value distribution and µ and σ are the model´s parameters.

Threshold estimations were calculated for 50% discrimination ^32^.

To estimate percent discrimination probability versus concentration distribution, data were performed in R version 4.0.0 Statistical package (The R Foundation for Statistical Computing). The survreg function of the survival package was used.

## Results

### Calcium gluconate monohydrate

**Figure 1.**
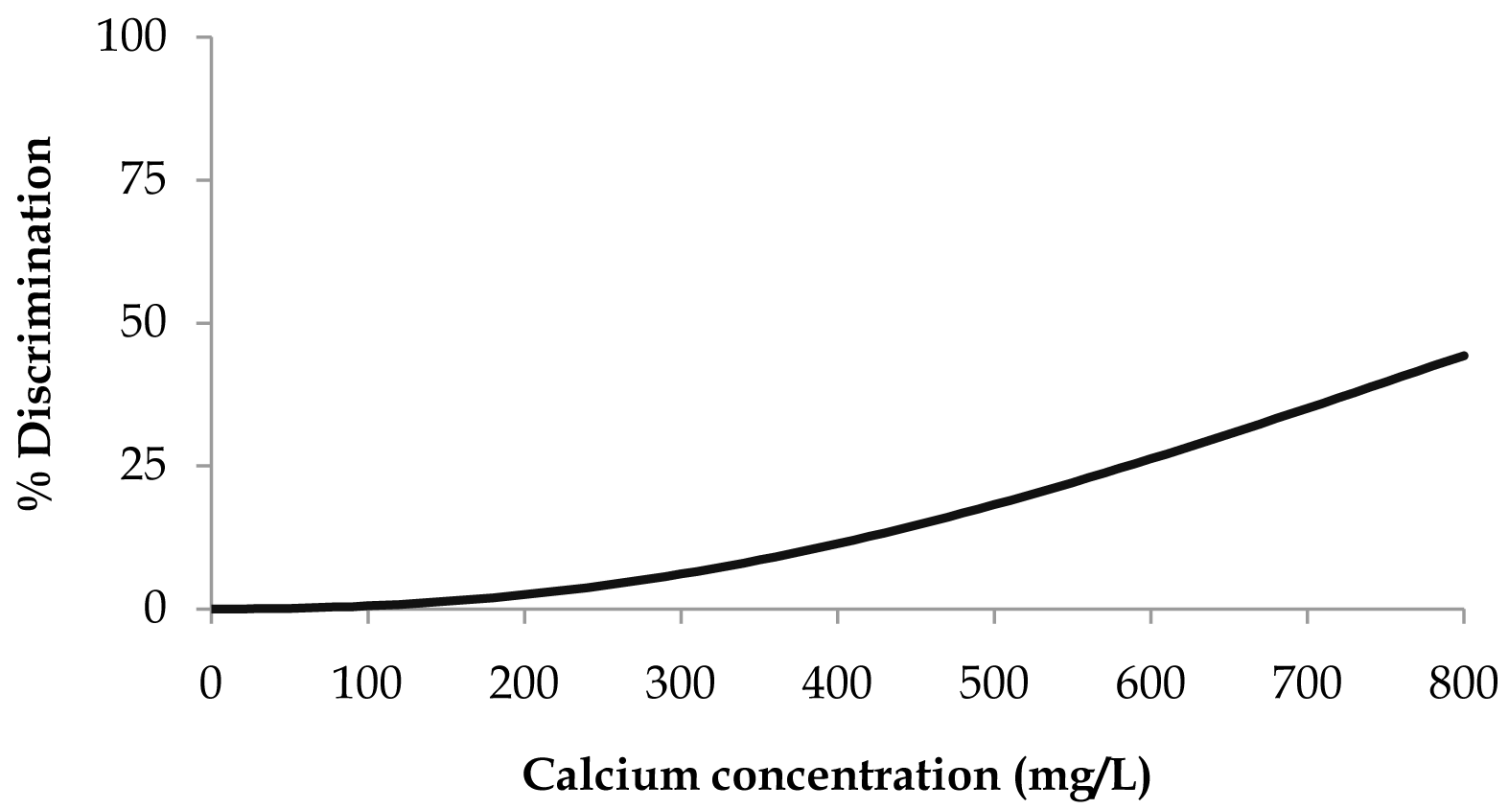
Calcium gluconate monohydrate threshold taste. Percent discrimination versus calcium concentration for the Weibull distribution.

When applying the survival analysis methodology to the calcium gluconate threshold data, the best fitting distribution was the Weibull ( Equation 2). The resulting parameters ± 95% confidence intervals were μ = 6.9 ± 0.2 and σ = 0.44 ± 0.15 ^33^. Percent discrimination versus concentration for this Weibull distribution is plotted in Figure 1.

The threshold value estimation corresponding to 25% discrimination ± 95% confidence intervals was 587 ± 131 mg of Ca/L, corresponding to a water sample with a calcium gluconate concentration of 6.6 ± 1.4 g/L.

We were not able to estimate the threshold value estimation corresponding to 50% discrimination as the maximum number of successful answers obtained from the consumer panel reached 44%.

### Calcium lactate pentahydrate

**Figure 2.**
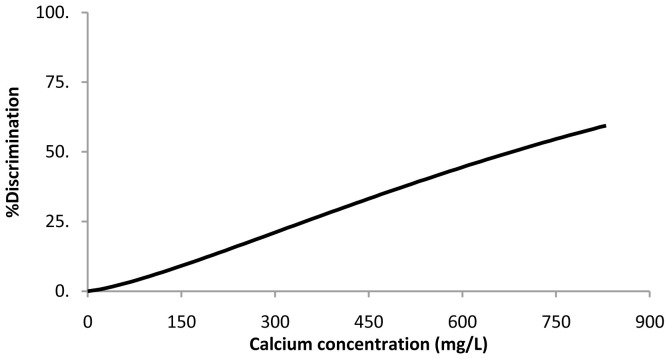
Calcium lactate pentahydrate threshold taste. Percent discrimination versus calcium concentration for the Weibull distribution.

For the calcium lactate threshold, the Weibull was the best fitting distribution ( Equation 2). The resulting parameters ± 95% confidence intervals were μ = 6.8 ± 0.3 and σ = 0.76 ± 0.25. Percent discrimination versus concentration for this Weibull distribution is plotted in Figure 2. The threshold value estimation corresponding to 50% discrimination ± 95% confidence intervals was 676 ± 186 mg of Ca/L, corresponding to a water sample with a calcium lactate concentration of 5.2 ± 1.4 g/L.

### Calcium chloride dihydrate

**Figure 3.**
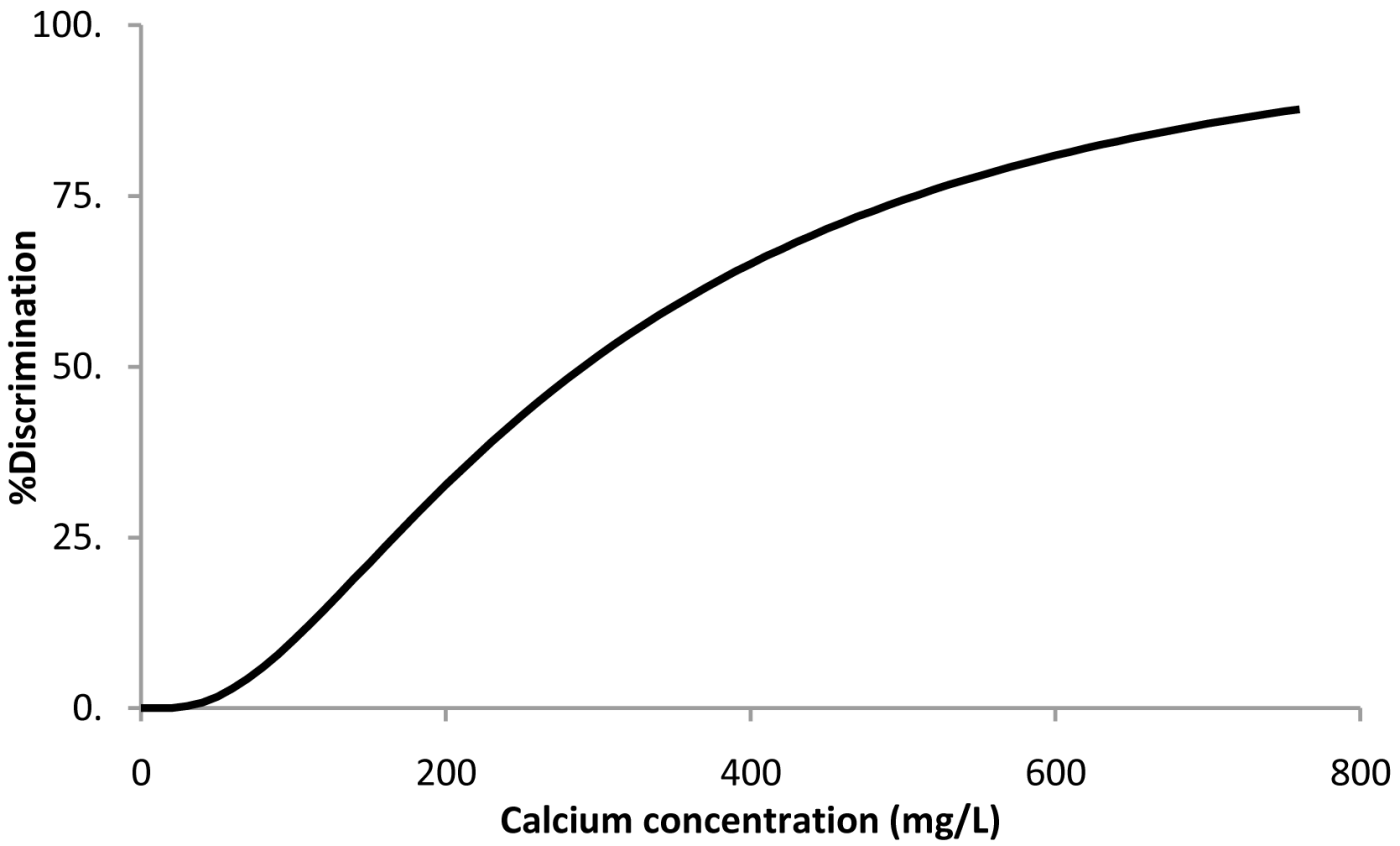
Calcium chloride dihydrate threshold taste. Percent discrimination versus calcium concentration for the log normal distribution.

When the survival analysis methodology was applied to the calcium chloride threshold data, the best fitting distribution was the log-normal ( Equation 1). The resulting parameters ± 95% confidence intervals were μ = 5.7 ± 0.3 and σ = 0.83 ± 0.20. Percent discrimination versus concentration for this log-normal distribution is plotted in Figure 3.

The threshold value estimation corresponding to 50% discrimination ± 95% confidence intervals was 291 ± 73 mg of Ca/L, corresponding to a water sample with a calcium chloride concentration of 1.1 ± 0.3 g/L.

## Discussion

This study shows that the sensory detection threshold of water with added calcium salts allows the increase of calcium concentration of water up to a level of 500 mg of calcium /L. The feasibility of using water with added calcium to improve dietary intake will depend on the drinking water distribution system, which will define the type of salt and concentration to be used. Inorganic salts such as calcium chloride could be used to increase the calcium content of bottled or tap water. Further tests should be done in order to determine the maximum level that could be added to tap water while complying with regulations for tap drinking water. On the other hand, organic salts such as calcium gluconate and calcium lactate can only be used to increase the calcium concentration of bottled drinks, and their application needs further studies on safety and stability. Further studies should also be performed to establish shelf life.

Considering that most drinking tap and bottled waters have very low calcium concentrations, the level of calcium attained in this study would involve a significant increase to impact calcium intake at population level ^13, 17^. In this study, it was possible to define the threshold for taste using the triangle test methodology and survival analysis statistics.

Alcaire *et al.* (2014) applied survival analysis to estimate equivalent sweet concentration of low-calorie sweeteners in orange juice ^34^. They found its main advantage is the consideration of individual differences among assessors, which may lead to more accurate estimations than those obtained with other methodologies.

Reis *et al.* (2016) compared two sensory methodologies (paired comparison and magnitude estimation) and two data analysis approaches (logistic regression and survival analysis) to estimate equivalent sweet concentration of high-intensity sweeteners ^35^. They found paired comparison and magnitude estimation provided similar estimations for the sweeteners, but logistic regression and survival analysis differed in the accuracy of the estimations. Data analysis performed using survival analysis gave more accurate estimations.

The study followed a standardized methodology and analysis of survival for measurements that took into account answers given by chance. The sensory discrimination test is easy to perform and understand for assessors ^34^.

One limitation of this study is that the panel of water consumers was all from a town in Argentina where calcium concentrations in drinking water are below 50 mg/L. Therefore, if this strategy is intended to be applied in populations with different water composition, the same test would need to be replicated. Another limitation is that solutions were prepared the previous day; further studies should test solution stability for longer periods of time to assess if there is any precipitation.

## Conclusion

These results show that it is feasible to obtain water with added calcium using different salts and reach a concentration of up to 500 mg of calcium/L of water. The calcium salt allowing the highest calcium concentration with the lowest perceived changes in taste was calcium gluconate. Future studies need to explore stability and acceptability over longer periods of time.

## Data availability

### Underlying data

Mendeley Data: Water with added calcium. https://doi.org/10.17632/9fj6fs7kf2.1 ^33^

Data are available under the terms of the Creative Commons Attribution 4.0 International license (CC-BY 4.0).
