# Supplementary material for: Testing for sensory threshold in drinking water with added calcium: a first step towards developing a calcium fortified water
Source: Gates Open Res. 2022 Jan 10;5:151. Originally published 2021 Oct 19. [Version 2] doi: 10.12688/gatesopenres.13361.2 (PMC8753188; doi:10.12688/gatesopenres.13361.2)
Supplement: Peer review history

# Peer review: Reviewer response for version 2

**DOI:** 10.21956/gatesopenres.14755.r31602

**Published:** 2022-01-11

**Authors:** Megan W. Bourassa, Ziaul Rana

**Affiliations:** 1 Nutrition Science, The New York Academy of Sciences, New York City, NY, USA; 2 New York Academy of Sciences, New York City, NY, USA

Thank you for the responses to the comments. They have very much helped answer my questions.

Is the work clearly and accurately presented and does it cite the current literature?

Yes

If applicable, is the statistical analysis and its interpretation appropriate?

I cannot comment. A qualified statistician is required.

Are all the source data underlying the results available to ensure full reproducibility?

Yes

Is the study design appropriate and is the work technically sound?

Yes

Are the conclusions drawn adequately supported by the results?

Yes

Are sufficient details of methods and analysis provided to allow replication by others?

Partly

Reviewer Expertise:

Nutrition science, micronutrients, biochemistry.

We confirm that we have read this submission and believe that we have an appropriate level of expertise to confirm that it is of an acceptable scientific standard.

# Peer review: Reviewer response for version 1

**DOI:** 10.21956/gatesopenres.14606.r31276

**Published:** 2021-11-22

**Authors:** Erick Boy

**Affiliations:** 1 HarvestPlus, International Food Policy Research Institute, Washington, DC, USA

The study of widely consumed vehicles (foods, condiments and beverages) to delivery nutrients that are not adequately present in sufficient bioavailable amounts in a population's typical diet is an important public health measure. Although the importance of calcium by itself to prevent pregnancy induced hyptertension, high blood pressure in general, or osteoporosis has not been adequately studied, the low supply of calcium in most of the LMIC is a fact that has been reproduced by different researchers using relatively different methodologies. Drinking water is an attractive potential vehicle for minerals like Ca, Zn and Fe. This is a proof of concept investigation for delivering calcium using three likely calcium salts regarding solubility and organoleptic effects of gluconate, lactate, and chloride).

 Regarding applicability of results, it would have been useful to model the cost of this intervention with calcium chloride using a hypothetical water supply system or an organic salt for bottled water (and adjusting results for water utilization for purposes other than drinking/cooking, e.g., wastage).  Although survival analysis is more often used for acceptability during shelf life studies, it is adequate for acceptability testing, which uses 9 point Hedonic scales more often. Given the relative simplicity and low cost of the methodology used, it seems important for increasing the external validity of results to conduct similar experiments in other populations (e.g., India or Bangladesh). The long term effects of increased calcium content on delivery systems and on human health should also be discussed. The potential risk of renal load when using calcium levels above 200mg/L for young children and labeling implications for bottled water should be discussed as well.

Is the work clearly and accurately presented and does it cite the current literature?

Yes

If applicable, is the statistical analysis and its interpretation appropriate?

I cannot comment. A qualified statistician is required.

Are all the source data underlying the results available to ensure full reproducibility?

Yes

Is the study design appropriate and is the work technically sound?

Yes

Are the conclusions drawn adequately supported by the results?

Yes

Are sufficient details of methods and analysis provided to allow replication by others?

Yes

Reviewer Expertise:

Human nutrition, micronutrients (iron, zinc, vitamin A, iodine), biofortification, vitamin A fortification of sugar, iodine fortification of salt, food fortification.

I confirm that I have read this submission and believe that I have an appropriate level of expertise to confirm that it is of an acceptable scientific standard.

# Peer review: Reviewer response for version 1

**DOI:** 10.21956/gatesopenres.14606.r31280

**Published:** 2021-11-15

**Authors:** Megan W. Bourassa, Ziaul Rana

**Affiliations:** 1 Nutrition Science, The New York Academy of Sciences, New York City, NY, USA; 2 New York Academy of Sciences, New York City, NY, USA

This manuscript determined the sensory threshold of different calcium salts added to drinking water using survival analysis. It provides a new perspective and a mechanism for improving calcium intake through fortification.

 Their methodological approach is good, and it is a well-written manuscript. It should be indexed once some minor edits are made.

- Include information on purity of the salts and if they were food-grade.
- An explanation of the judge panel, particularly their experience, would have been helpful. What was the range of concentrations provided to the expert panel?  
- The test samples were labeled with a 3-digit code to blind the consumer panel but were the study administrators also blinded?
- An experiment with a case-control design would have provided more experimental reliability. It would help us understand how fortified water differs from regular water in terms of sensory quality.
- A description of the water's quality, such as its natural hardness, is essential. As it is written it is unclear how much calcium is in the water before the experimental calcium is added. It states calcium 27 mg/L, alkalinity (CaCO3) 80 mg/L, hardness (CaCO3) 104 mg/L. What is the total amount of calcium already present?
- More information on the tasting order and procedure would be helpful. How many different samples did participants receive? If each batch contained 2 identical and one different sample, and they received a total of 7 batches over 2 days, it seems like they did not get to taste all of the solutions. Was each batch different, did the participants test only one salt form or did they get all three?
- How do these findings compare with the organoleptic and sensory tests done previously for calcium (as mentioned in the introduction)?

Is the work clearly and accurately presented and does it cite the current literature?

Yes

If applicable, is the statistical analysis and its interpretation appropriate?

I cannot comment. A qualified statistician is required.

Are all the source data underlying the results available to ensure full reproducibility?

Yes

Is the study design appropriate and is the work technically sound?

Yes

Are the conclusions drawn adequately supported by the results?

Yes

Are sufficient details of methods and analysis provided to allow replication by others?

Partly

Reviewer Expertise:

Nutrition science, micronutrients, biochemistry.

We confirm that we have read this submission and believe that we have an appropriate level of expertise to confirm that it is of an acceptable scientific standard, however we have significant reservations, as outlined above.

# Response

**Published:** 2021-12-14

**Authors:** Gabriela Cormick

**Affiliations:** CONICET, Argentina

**Response to reviewers**

**APPROVED WITH RESERVATIONS**

 This manuscript determined the sensory threshold of different calcium salts added to drinking water using survival analysis. It provides a new perspective and a mechanism for improving calcium intake through fortification.

  Their methodological approach is good, and it is a well-written manuscript. It should be indexed once some minor edits are made.

1. Include information on purity of the salts and if they were food-grade.

***ANSWER: Thank you for your remark, we have included the specification given by the provider to reinforce that the salts were suitable for human consumption.***

***Material and Methods:*** *“* *Calcium chloride dihydrate was purchased from Sigma-Aldrich Corporation* *and meets analytical specifications of The European Pharmacopoeia (Ph. Eur.), Pharmaceutical Reference Standards (USP), FCC, E509, whereas calcium lactate pentahydrate and calcium gluconate Monohydrate were purchased from Surfactan, both meet analytical specifications of USP and European Pharmacopoeia (EP).* *”*

1. An explanation of the judge panel, particularly their experience, would have been helpful. What was the range of concentrations provided to the expert panel?  

***ANSWER: Thank you for this comment, we have now included information on the process in the section: “*** **Selection of salt and concentration” (page 3):**

***Material and Methods: *** “The expert panel compared different concentrations of each salt against water without added calcium. Using a triangle test the range was set from a concentration where no sensory differences were detected to a concentration where marked differences were detected. After 7 triangular sessions the panel set the range between 77 and 800 mg of Ca / L for calcium chloride, 107 and 820 mg of Ca / L for calcium lactate, and 107 and 863 mg of Ca / L for calcium gluconate (Table 1). “

1. The test samples were labeled with a 3-digit code to blind the consumer panel but were the study administrators also blinded?

***ANSWER: We have included the following details of the blinding process.***

***Material and Methods: “*** following sensory analysis protocols, where only participants are blinded to the samples.[32] (Lawless et al. 2010)”

**Lawless, Harry T. T. 2nd ed. 2010. Sensory Evaluation of Food: Principles and Practices. ** 

1. An experiment with a case-control design would have provided more experimental reliability. It would help us understand how fortified water differs from regular water in terms of sensory quality.

**Answer: **Thank you for this valuable comment. In this study we only tested the sensory threshold, following you suggestion further studies should be performed to assess the acceptability of the water in the long term.

1. A description of the water's quality, such as its natural hardness, is essential. As it is written it is unclear how much calcium is in the water before the experimental calcium is added. It states calcium 27 mg/L, alkalinity (CaCO3) 80 mg/L, hardness (CaCO3) 104 mg/L. What is the total amount of calcium already present?

***Material and Methods:***

*“Baseline water*

*All the concentrations prepared from the different calcium salts were tested against a reference table bottled water that is commonly consumed in the country and complies with the standards of the water cooperative in Argentina (Instituto verificador de elaboración de soda en sifones, IVESS). [30] According to the information provided by the cooperative, this water is filtered, dechlorinated, ozone purified tap water. The baseline bottled water has a calcium concentration of 27 mg/L and water hardness of 104 mg/L (CaCO3). This water also contained 32 mg/L of sodium, 10mg/L of nitrates, 38 mg/L of chlorides, 80 mg/L of alkalinity (CaC03) and 270 mg/L total dissolved solids.*

*Samples preparation*

*Samples were prepared using the previously described baseline table bottled water ….”*

  

1. More information on the tasting order and procedure would be helpful. How many different samples did participants receive? If each batch contained 2 identical and one different sample, and they received a total of 7 batches over 2 days, it seems like they did not get to taste all of the solutions. Was each batch different, did the participants test only one salt form or did they get all three?

***ANSWER:*** *Thank you for highlighting this, we have reordered the section and added more information on the methodology* *.*

  “After the training, threshold tests were carried out. In this test, a batch of three samples were presented simultaneously to the panelists, two samples were from the same concentration and one  from a different concentration. Each panelist had to indicate which sample was the odd sample. There were six possible serving orders (AAB, ABA, BAA, BBA, BAB, ABB) which were counterbalanced across all panelists. Consumers were asked to taste each sample in the row (left to right) and to select the odd sample. Between batches, participants were asked to neutralize taste with mineral water and white bread.

 Each consumer received seven batches of three 30 ml solutions. The tests were performed on different days to avoid tiredness or flavour carryover. For each salt, consumers tested three batches one day and the remaining four batches on a second day.”

 Samples were presented at room temperature (18–23°C) in similar polystyrene 70 mL cups coded with three-digit random to allow blinding. Following the sensory analysis protocol, participants were blinded to the samples however the panel leader and assistant were not.[32]

  

1. How do these findings compare with the organoleptic and sensory tests done previously for calcium (as mentioned in the introduction)?

***ANSWER:***

 The studies mentioned in the introduction are based on simulations to assess the effectiveness of calcium fortified water to decrease calcium inadequate intake. However, these simulations did not consider the sensory threshold of water with added calcium. As we mention in the discussion, this study shows that the sensory detection threshold of water with added calcium salts allows the increase of calcium concentration of water up to a level of 500 mg of calcium /L which is the value we have shown it would improve calcium intakes without posing any risk of calcium excess to the population. 

 Discussion: “This study shows that the sensory detection threshold of water with added calcium salts allows the increase of calcium concentration of water up to a level of 500 mg of calcium /L. “

 Second reviewer: Boy Erick

 Thank you for these valuable comments that will orient further studies of our group.
